# Analysis of septins across kingdoms reveals orthology and new motifs

**DOI:** 10.1186/1471-2148-7-103

**Published:** 2007-07-01

**Authors:** Fangfang Pan, Russell L Malmberg, Michelle Momany

**Affiliations:** 1 Plant Biology Department, University of Georgia, Athens, GA, 30602, USA

## Abstract

**Background:**

Septins are cytoskeletal GTPase proteins first discovered in the fungus *Saccharomyces cerevisiae *where they organize the septum and link nuclear division with cell division. More recently septins have been found in animals where they are important in processes ranging from actin and microtubule organization to embryonic patterning and where defects in septins have been implicated in human disease. Previous studies suggested that many animal septins fell into independent evolutionary groups, confounding cross-kingdom comparison.

**Results:**

In the current work, we identified 162 septins from fungi, microsporidia and animals and analyzed their phylogenetic relationships. There was support for five groups of septins with orthology between kingdoms. Group 1 (which includes *S. cerevisiae *Cdc10p and human Sept9) and Group 2 (which includes *S. cerevisiae *Cdc3p and human Sept7) contain sequences from fungi and animals. Group 3 (which includes *S. cerevisiae *Cdc11p) and Group 4 (which includes *S. cerevisiae *Cdc12p) contain sequences from fungi and microsporidia. Group 5 (which includes *Aspergillus nidulans *AspE) contains sequences from filamentous fungi. We suggest a modified nomenclature based on these phylogenetic relationships. Comparative sequence alignments revealed septin derivatives of already known G1, G3 and G4 GTPase motifs, four new motifs from two to twelve amino acids long and six conserved single amino acid positions. One of these new motifs is septin-specific and several are group specific.

**Conclusion:**

Our studies provide an evolutionary history for this important family of proteins and a framework and consistent nomenclature for comparison of septin orthologs across kingdoms.

## Background

Septins were first identified in the budding yeast *Saccharomyces cerevisiae *where they have been very well-characterized [1]. In *S. cerevisiae *five septins, Cdc3p, Cdc10p, Cdc11p, Cdc12p and Shs1p, polymerize to form a ring at the mother-bud neck where they are important for bud site selection and cytokinesis. Two other yeast septins, Spr3p and Spr28p, are expressed during sporulation [2,3]. Yeast septins have been shown to function as a scaffold organizing the division site and coordinating nuclear and cellular division. Septins have also been shown to act as a barrier, preventing diffusion of RNA and proteins between mother and daughter cells [1,4]. Though not as well-characterized as those in yeast, septins in other fungi also appear to organize sites of cell division and new growth [5]. Septins have been found in a variety of animal tissues. In addition to acting as a diffusion barrier, animal septins are implicated in vesicle trafficking, apoptosis and cell movement [6]. In mammals septins appear to regulate membrane and cytoskeleton organization and abnormal septins have been linked with cancer and neurodegeneration [7-9].

Septins are P-loop GTPase proteins [10]. P-loop GTPases, including kinesin, myosin and ras proteins share at least five conserved motifs designated G1 to G5 within the GTP-binding domain [11]. The G1 motif, defined by the consensus element GxxxxGK [ST], forms a flexible loop which interacts with the phosphate group of the nucleotide [10-12]. The G2 motif is conserved within individual GTPase families, but not across the whole class [11]. The G3 motif contains several hydrophobic residues followed by DxxG [10-12]. This region binds Mg^2+ ^and can interact with β and γ phosphates of GTP [10,11,13]. The G4 motif, NKxD, is important for GTP binding specificity [10,14]. The G5 motif is found in some, but not all, members of the P-loop GTPase class [11].

**Figure 1:**
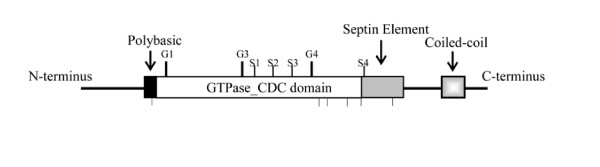
**Typical septin structure**. Septin sequences range from about three hundred to six hundred amino acids. Septins contain the conserved GTP_CDC binding domain with three motifs: G1, GxxxxGK [ST] (amino acids 126–135 in *S. cerevisiae *Cdc3p); G3, DxxG (amino acids 204–209 in *S. cerevisiae *Cdc3p); and G4, xKxD (amino acids 280–289 in *S. cerevisiae *Cdc3p). The previously described polybasic region (amino acids 110–120 in *S. cerevisiae *Cdc3p; [19, 21]) is shown as a black box and the previously described "septin unique element" (amino acids 360–413 in *S. cerevisiae *Cdc3p [21]); is shown as a grey box. S1-S4 mark positions of new septin motifs (Table 2b; amino acid 237–242, 247–259, 261–268, 364–365 in *S. cerevisiae *Cdc3p) and lines below diagram show conserved single amino acid positions (Table 2c; amino acids 117, 295, 300, 339, 360, 396 in *S. cerevisiae *Cdc3p). Many septins also have a predicted coiled-coil domain at the C-terminus (amino acids 476–507 in *S. cerevisiae *Cdc3p; [21]).

Septins clearly contain the G1, G3 and G4 motifs [15] (Figure 1). Septins purified from *Drosophila*, *Xenopus *and *Saccharomyces *have been shown to bind or hydrolyze GTP though the biological significance of these activities and the specific functions of these motifs are not yet clear [16-18]. N-terminal to the GTPase domain, septins contain a polybasic region that has been shown to bind phosphoinositides [19,20]. C-terminal to the GTPase domain, a 53 amino acid septin element conserved among many septins has been previously identified [21]. Most septins also contain a C-terminal extension predicted to form coiled-coils and shown to be needed for interactions between certain septins [19,22,23].

Previously fungal septins were placed into groups based on phylogenetic analysis [24] and mammalian septins were placed into groups based on primary sequence similarity [6]. Kinoshita [25] used phylogenetic analysis of two fungal yeast species and three animal species to conclude that orthologous relationships existed within fungal or animal septins, but not between fungal and animal septins making it impossible to compare model fungi and less tractable animals [25]. Recent genome projects provide an excellent opportunity to better understand the evolutionary relationships of septins. Here we identify 162 septins from 36 fungi, microsporidia and animals. Based on phylogenetic analysis we place the septins into five groups, two of which clearly contain orthologous fungal and animal septins. We also present three modified GTPase motifs, four new motifs and six individual amino acid positions which have been conserved among fungal, microsporidial and animal septins. Our results suggest that it should be possible to apply lessons learned from a subset of septins in model organisms to septins from mammals.

## Results

### Database searches identified 166 septin-related sequences

We used the Cdc3p sequence of *Saccharomyces cerevisiae*, one of the best-studied septins, to query GenBank with the PSI-BLAST program and detected 876 sequences. From the PSI-BLAST list we identified 166 unique potential septin sequences based on an e-value lower than e^-3^, the presence of the G1, G3 and G4 GTPase motifs and other sequence similarities (Table 1). In our designation, the first three letters represent the species from which the sequence came (e.g. Sce represents *Saccharomyces cerevisiae*). Three septin sequences appeared to be truncated and were eliminated from further consideration (AbiSep, DyaSep2, and ZroCDC). We individually checked each of the remaining 163 potential septin sequences for the presence of the GTP_CDC domain [26].

**Table 1:** Septin and septin-like sequences analyzed.

GI*	**Name in Paper^†^**	Proposed Name*	**Alias^‡^**	**Clade/Species^§^**		**Group^¶^**	**G^||^**	C**
gi|2244629	AbiSep	/	sepA	F	Agaricus bisporus	Tr	+	+
gi|31198659	AgaHyp1	Pnut		A	Anopheles gambiae	2B	+	+
gi|31202059	AgaHyp2	Sep2		A	Anopheles gambiae	1B	+	+
gi|31206631	AgaHyp3	Sep1		A	Anopheles gambiae	2B	+	+
gi|31204715	AgaHyp4	Sep4		A	Anopheles gambiae	2B	+	+
gi|13398364	AniAspA	Cdc11		F	Aspergillus nidulans	3	+	+
gi|1791305	AniAspB	Cdc3		F	Aspergillus nidulans	2A	+	+
gi|34811845	AniAspC	Cdc12		F	Aspergillus nidulans	4	+	+
gi|34148975	AniAspD	Cdc10		F	Aspergillus nidulans	1A	+	-
gi|34811843	AniAspE	AspE		F	Aspergillus nidulans	5	+	-
gi|45645169	BtaCdc10	/		A	Bos taurus	2B	+	+
gi|39580970	CbrHyp1	Unc59b	CBG20268	A	Caenorhabditis briggsae	2B	+	+
gi|39589843	CbrHyp2	Unc61	CBG04550	A	Caenorhabditis briggsae	1B	+	+
gi|39584450	CbrHyp3	Unc59a	CBG19777	A	Caenorhabditis briggsae	2B	+	+
gi|17509405	CelUnc59	/		A	Caenorhabditis elegans	2B	+	+
gi|32566810	CelUnc61	/		A	Caenorhabditis elegans	1B	+	+
gi|729090	CalCdc3	/		F	Candida albicans	2A	+	+
gi|729064	CalCdc10	/		F	Candida albicans	1A	+	-
gi|21435770	CalCdc11	/		F	Candida albicans	3	+	+
gi|21435778	CalCdc12	/		F	Candida albicans	4	+	+
gi|21435802	CalSep7	Shs1	SHS1	F	Candida albicans	3	+	+
gi|46442449	CalSpr28	/		F	Candida albicans	3	+	-
gi|46444553	CalSpr3	/		F	Candida albicans	4	+	+
gi|50286825	CglHyp1	Cdc3		F	Candida glabrata	2A	+	+
gi|50284895	CglHyp2	Cdc12a		F	Candida glabrata	4	+	+
gi|50288449	CglHyp3	Cdc10		F	Candida glabrata	1A	+	-
gi|50289749	CglHyp4	Cdc12b		F	Candida glabrata	4	+	+
gi|50287113	CglHyp5	Cdc11		F	Candida glabrata	3	+	+
gi|50288341	CglHyp6	Shs1		F	Candida glabrata	3	+	+
gi|50291973	CglHyp7	Spr3		F	Candida glabrata	4	+	+
gi|50286937	CglHyp8	Spr28		F	Candida glabrata	3	+	+
gi|18476091	CimSep1	Cdc11		F	Coccidioides immitis	3	+	+
gi|24637104	CimSep2	Cdc3		F	Coccidioides immitis	2A	+	+
gi|24637108	CimSep3	Cdc10		F	Coccidioides immitis	1A	+	-
gi|24473756	CimSep4	AspE		F	Coccidioides immitis	5	+	-
gi|50257384	CneHyp1	Cdc3		F	Cryptococcus neoformans	2A	+	+
gi|50259101	CneHyp2	Cdc12		F	Cryptococcus neoformans	4	+	+
gi|50258769	CneHyp3	Cdc10		F	Cryptococcus neoformans	1A	+	-
gi|50257720	CneHyp4	Cdc11		F	Cryptococcus neoformans	3	+	+
gi|50260201	CneHyp5	AspE		F	Cryptococcus neoformans	5	+	-
gi|41055580	DreHyp1	Sept7	zgc:56383	A	Danio rerio	2B	+	+
gi|32822794	DreHyp2	Sept8	wu:fb22a03	A	Danio rerio	1B	+	+
gi|41152396	DreHyp4	Sept5	zgc:73218	A	Danio rerio	2B	+	+
gi|40538786	DreMsf	Sept9	MLL septin-like fusion	A	Danio rerio	1A	+	-
gi|45709377	DreNedd5	Sept2	zgc:63587	A	Danio rerio	2B	+	+
gi|47086783	DreSept6	/	zgc:66071	A	Danio rerio	1B	+	+
gi|50420949	DhaHyp1	Cdc3		F	Debaryomyces hansenii	2A	+	+
gi|50426961	DhaHyp2	Cdc12		F	Debaryomyces hansenii	4	+	+
gi|50425027	DhaHyp3	Cdc10		F	Debaryomyces hansenii	1A	+	-
gi|50426163	DhaHyp4	Spr3		F	Debaryomyces hansenii	4	+	+
gi|50418421	DhaHyp5	Cdc11		F	Debaryomyces hansenii	3	+	+
gi|50414330	DhaHyp6	Shs1		F	Debaryomyces hansenii	3	+	+
gi|730352	DmePnut	/		A	Drosophila melanogaster	2B	+	+
gi|17647925	DmeSep1	/		A	Drosophila melanogaster	2B	+	+
gi|17738071	DmeSep2	/		A	Drosophila melanogaster	1B	+	+
gi|24642597	DmeSep4	/	CG9699	A	Drosophila melanogaster	2B	+	+
gi|21356243	DmeSep5	/		A	Drosophila melanogaster	1B	+	+
gi|38047705	DyaSep2	/		A	Drosophila yakuba	Tr	-	+
gi|19075150	EcuSep1	Spr3a	ECU01_1370	M	Encephalitozoon cuniculi	4	+	+
gi|19074995	EcuSep2	Spr3b	ECU11_1950	M	Encephalitozoon cuniculi	4	+	+
gi|19173204	EcuSep3	Cdc11	ECU09_0820	M	Encephalitozoon cuniculi	3	+	-
gi|45198629	EgoHyp1	Cdc3		F	Eremothecium gossypii	2A	+	+
gi|45190841	EgoHyp2	Cdc12		F	Eremothecium gossypii	4	+	+
gi|45184824	EgoHyp3	Cdc10		F	Eremothecium gossypii	1A	+	-
gi|45191046	EgoHyp4	Cdc11		F	Eremothecium gossypii	3	+	+
gi|45199089	EgoHyp5	Spr3		F	Eremothecium gossypii	4	+	+
gi|45201271	EgoHyp6	Spr28		F	Eremothecium gossypii	3	+	-
gi|45185071	EgoHyp7	Shs1		F	Eremothecium gossypii	3	+	+
gi|14041182	GcySep1	/		A	Geodia cydonium	2B	+	+
gi|29249771	Gla	/		P	Giardia lamblia	Slk	-	-
gi|46121875	GzeHyp1	Cdc3		F	Gibberella zeae	2A	+	+
gi|46126005	GzeHyp2	Cdc12		F	Gibberella zeae	4	+	+
gi|46135811	GzeHyp3	Cdc11		F	Gibberella zeae	3	+	+
gi|46123315	GzeHyp4	Cdc10		F	Gibberella zeae	1A	+	-
gi|46128665	GzeHyp5	AspE		F	Gibberella zeae	5	+	-
gi|46122029	GzeHyp6	AspE2		F	Gibberella zeae	5	+	-
gi|46139179	GzeHyp7	/		F	Gibberella zeae	Slk	+	+
gi|16604248	HsaSept1	/		A	Homo sapiens	2B	+	+
gi|4758158	HsaSept2	/	Nedd5, Pnutl3, Diff6, KIA0158	A	Homo sapiens	2B	+	+
gi|22035572	HsaSept3	/		A	Homo sapiens	1A	+	-
gi|4758942	HsaSept4	/	H5, Bradion, Pnutl2, ARTS, MART, hCDCrel-2, Septin-M	A	Homo sapiens	2B	+	+
gi|9945439	HsaSept5	/	Pnutl, hCDCrel-1	A	Homo sapiens	2B	+	+
gi|22035577	HsaSept6	/	Sept2, KIA0128	A	Homo sapiens	1B	+	+
gi|4502695	HsaSept7	/	hCdc10	A	Homo sapiens	2B	+	+
gi|41147049	HsaSept8	/	KIA0202	A	Homo sapiens	1B	+	+
gi|6683817	HsaSept9	/	AF17q25, MSF, SepD1, Ov/Br septin, Pnutl4, KIA0991	A	Homo sapiens	1A	+	-
gi|18088518	HsaSept10	/	Sep1-like	A	Homo sapiens	1B	+	+
gi|8922712	HsaSept11	/	FLJ10849	A	Homo sapiens	1B	+	+
gi|23242699	HsaSept12	/	FLJ25410	A	Homo sapiens	1A	+	-
gi|113418512	HsaSept13	/		A	Homo sapiens	2B	+	+
gi|50306547	KlaHyp1	Cdc3		F	Kluyveromyces lactis	2A	+	+
gi|50309827	KlaHyp2	Cdc12		F	Kluyveromyces lactis	4	+	+
gi|50311269	KlaHyp3	Cdc10		F	Kluyveromyces lactis	1A	+	-
gi|50303889	KlaHyp4	Spr3		F	Kluyveromyces lactis	4	+	+
gi|50304439	KlaHyp5	Cdc11		F	Kluyveromyces lactis	3	+	+
gi|50311965	KlaHyp6	Shs1		F	Kluyveromyces lactis	3	+	+
gi|50311291	KlaHyp7	Spr28		F	Kluyveromyces lactis	3	+	-
gi|13358928	MfaHyp1	Sept5		A	Macaca fascicularis	2B	+	+
gi|38110101	MgrHyp1	Cdc3		F	Magnaporthe grisea	2A	+	+
gi|38106951	MgrHyp2	Cdc12		F	Magnaporthe grisea	4	+	+
gi|38109157	MgrHyp3	Cdc10		F	Magnaporthe grisea	1A	+	-
gi|38100755	MgrHyp4	Cdc11		F	Magnaporthe grisea	3	+	+
gi|38100224	MgrHyp5	AspE		F	Magnaporthe grisea	5	+	-
gi|38110686	MgrHyp6	AspE2		F	Magnaporthe grisea	5	+	-
gi|6453576	MciSepA	/	sepA	F	Mucor circinelloides	4	+	+
gi|8567344	MmuSept1	/	Diff6	A	Mus musculus	2B	+	+
gi|6754816	MmuSept2	/	Nedd5	A	Mus musculus	2B	+	+
gi|6755468	MmuSept3	/	mKIAA0991, G septin	A	Mus musculus	1A	+	-
gi|6755120	MmuSept4	/	M-Septin, H5	A	Mus musculus	2B	+	+
gi|6685763	MmuSept5	/	Cdcrel-1, Pnutl1	A	Mus musculus	2B	+	+
gi|20178348	MmuSept6	/		A	Mus musculus	1B	+	+
gi|9789726	MmuSept7	/	Cdc10	A	Mus musculus	2B	+	+
gi|39930477	MmuSept8	/	mKIAA0202	A	Mus musculus	1B	+	+
gi|28204888	MmuSept9	/	Sint1, E-septin, SLP-a	A	Mus musculus	1A	+	-
gi|26345492	MmuSept10a	/		A	Mus musculus	1B	+	+
gi|38082026	MmuSept10b	/	1700016K13Rik	A	Mus musculus	1B	+	+
gi|26324430	MmuSept11	/	D5Ertd606e	A	Mus musculus	1B	+	+
gi|20891621	MmuSept12	/	4933413B09Rik	A	Mus musculus	1A	+	-
gi|32417050	NcrHyp1	Cdc3		F	Neurospora crassa	2A	+	+
gi|32404966	NcrHyp2	Cdc12		F	Neurospora crassa	4	+	+
gi|32404320	NcrHyp3	Cdc10		F	Neurospora crassa	1A	+	-
gi|32422439	NcrHyp4	Cdc11		F	Neurospora crassa	3	+	+
gi|32417420	NcrHyp5	AspE		F	Neurospora crassa	5	+	-
gi|32411577	NcrHyp6	AspE2		F	Neurospora crassa	5	+	-
gi|32411845	NcrHyp7			F	Neurospora crassa	Slk	-	-
gi|5725417	PbrPbs1	Cdc11	pcd1	F	Pyrenopeziza brassicae	3	+	+
gi|34859284	RnoSept1	/	LOC293507	A	Rattus norvegicus	2B	+	+
gi|16924010	RnoSept2	/		A	Rattus norvegicus	2B	+	+
gi|9507085	RnoSept3	/	G-septin	A	Rattus norvegicus	1A	+	-
gi|32423788	RnoSept4	/	LOC287606, EG3RVC, EG3-1RVC	A	Rattus norvegicus	2B	+	+
gi|16758814	RnoSept5	/	Gp1bb, CDCrel-1, PNUTL1ai, CDCrel-1AI	A	Rattus norvegicus	2B	+	+
gi|34932994	RnoSept6	/	LOC298316	A	Rattus norvegicus	1B	+	+
gi|12018296	RnoSept7	/	Cdc10	A	Rattus norvegicus	2B	+	+
gi|34870727	RnoSept8	/	LOC303135	A	Rattus norvegicus	1B	+	+
gi|13929200	RnoSept9	/	Slpa, E-Septin	A	Rattus norvegicus	1A	+	-
gi|34882181	RnoSept10a	/	LOC309891	A	Rattus norvegicus	1B	+	+
gi|34872099	RnoSept10b	/	LOC288622	A	Rattus norvegicus	1B	+	+
gi|34876531	RnoSept11	/	LOC305227	A	Rattus norvegicus	1B	+	+
gi|34868752	RnoSept12	/	LOC363542	A	Rattus norvegicus	1A	+	-
gi|6323346	SceCdc3	/		F	Saccharomyces cerevisiae	2A	+	+
gi|6319847	SceCdc10	/		F	Saccharomyces cerevisiae	1A	+	-
gi|6322536	SceCdc11	/		F	Saccharomyces cerevisiae	3	+	+
gi|6321899	SceCdc12	/		F	Saccharomyces cerevisiae	4	+	+
gi|6319976	SceShs1	/	Sep7	F	Saccharomyces cerevisiae	3	+	+
gi|6320424	SceSpr28	/		F	Saccharomyces cerevisiae	3	+	+
gi|6321496	SceSpr3	/		F	Saccharomyces cerevisiae	4	+	+
gi|19115666	SpoSpn1	/		F	Schizosaccharomyces pombe	2A	+	+
gi|19114071	SpoSpn2	/		F	Schizosaccharomyces pombe	1A	+	-
gi|13638491	SpoSpn3	/		F	Schizosaccharomyces pombe	3	+	+
gi|19114478	SpoSpn4	/		F	Schizosaccharomyces pombe	4	+	+
gi|19114952	SpoSpn5	/		F	Schizosaccharomyces pombe	3	+	+
gi|19075714	SpoSpn6	/	SPCC584.09	F	Schizosaccharomyces pombe	4	+	+
gi|15214304	SpoSpn7	/	SPBC19F8.01c	F	Schizosaccharomyces pombe	3	+	-
gi|20177379	SdoSeptl	/		A	Suberites domuncula	2B	+	+
gi|33302067	UmaCdc10	/		F	Ustilago maydis	1A	+	-
gi|46099680	UmaHyp1	Cdc3		F	Ustilago maydis	2A	+	+
gi|46099269	UmaHyp2	Cdc12		F	Ustilago maydis	4	+	+
gi|46099354	UmaHyp3	Cdc11	Sep3	F	Ustilago maydis	3	+	+
gi|34784614	XlaHyp1	Sept12	MGC68931	A	Xenopus laevis	1A	+	-
gi|12003372	XlaSeptA	Sept2		A	Xenopus laevis	2B	+	+
gi|50551445	YliHyp1	Cdc3		F	Yarrowia lipolytica	2A	+	+
gi|50549207	YliHyp2	Cdc10		F	Yarrowia lipolytica	1A	+	-
gi|50551749	YliHyp3	Cdc12		F	Yarrowia lipolytica	4	+	+
gi|50553330	YliHyp4	Cdc11a		F	Yarrowia lipolytica	3	+	+
gi|50549013	YliHyp5	Spr28		F	Yarrowia lipolytica	3	+	+
gi|50547965	YliHyp6	Cdc11b		F	Yarrowia lipolytica	3	+	+
gi|50557032	YliHyp7	Spr3		F	Yarrowia lipolytica	4	+	+
gi|13940377	ZroCDC	/	er001-c	F	Zygosaccharomyces rouxii	Tr	+	-

* Genbank identification numbers.

† The first three letters represent genus and species names. The last letters represent current septin protein name.

* Proposed names based on first- or best-characterized septin in each clade.

‡ Alias designations from Genbank and [27, 28]

§ A represents animals; F represents fungi; M represents microsporidia.

**Figure 2:**
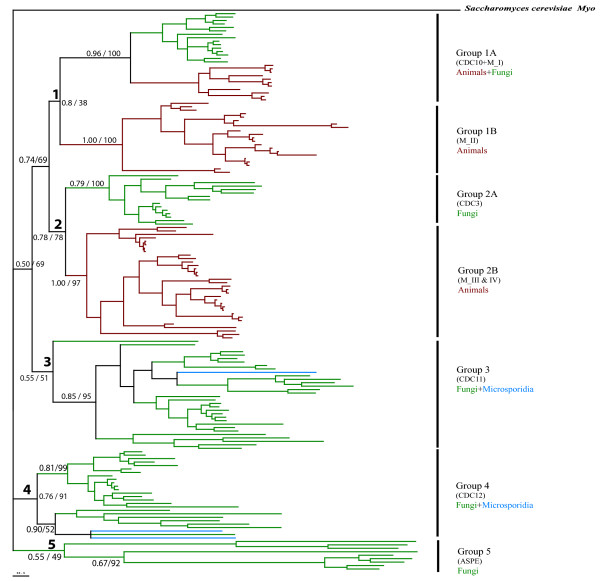
**Overview phylogenetic tree of septin gene family**. Half-compat consensus phylogram of 1.5 million generations of the MCMC analysis of the Bayesian phylogenetic analysis, discarding 400,000 generations as burn-in. Nodal numbers in front of the slash are posterior probabilities for Bayesian analysis. At the nodes where the tree topology agrees with the Bayesian analysis, numbers after the slash are bootstrap percentages from maximum likelihood bootstrap analysis using 1024 replicates. Red branches indicate animal lineage, green indicate fungal lineage and blue indicate microsporidia. Names in parenthesis under Group names indicate the best characterized fungal septin (CDC10, CDC3, CDC11 and CDC12, ASPE) or the mammalian septin classification of Martinez and Ware (2004) (MI, MII, MIII). See figures 3-5 for species names.

¶ Group names are assigned according to phylogenetic analysis shown in Figure 2. tr, truncated; slk, septin-like.

|| Presence of full length GTP_CDC detected by the SMART program.

** Predicted coiled-coil at C terminus.

Three of the 163 sequences were predicted to have only half of the GTP_CDC consensus domain and were designated "septin-like" (Gla, GzeHyp7 and NcrHyp7) (Table 1). In addition to septin sequences, our PSI-BLAST search with the Cdc3p query returned myosins and kinesins. A phylogenetic tree was built with representative septins, myosins, kinesins and ras GTPase family proteins to determine the relationship of the septin-like sequences to other GTPases. The three septin-like sequences did not group with any of the other GTPase superfamilies examined (data not shown). A BLAST search with the septin-like sequences did not give significant hits from any known protein families. This suggested that the septin-like sequences represent either ancient or diverged septins, or that they belong to an unknown protein family that shares some motifs with septins. The septin-like sequence found in *Giardia lamblia *is potentially illuminating for the evolution of this protein family because of *Giardia*'s position as a basal eukaryote.

The remaining 160 potential septin sequences grouped within a clade clearly separated from the other GTPase clades (data not shown). We designated these 160 sequences septins. After our PSI-BLAST search we became aware of two additional septins, human Sept 13 (HsaSept13) and *Ustilago maydis *Cdc10 (UmaCdc10), through reading of the literature [27,28]. We included these sequences for a total of 162 septins. Consistent with previous reports, we found septins in animals and fungi, but not in plants. Three septins were also found in the microsporidium Encephalitozoon cuniculi. We used a septin from *E. cuniculi *(EcuSepI, GenBank:gi|19075150) to query GenBank with PSI-BLAST a second time, and did not find any new potential septins.

### Phylogenetic Analysis

#### Bayesian analysis of all septins

**Figure 3:**
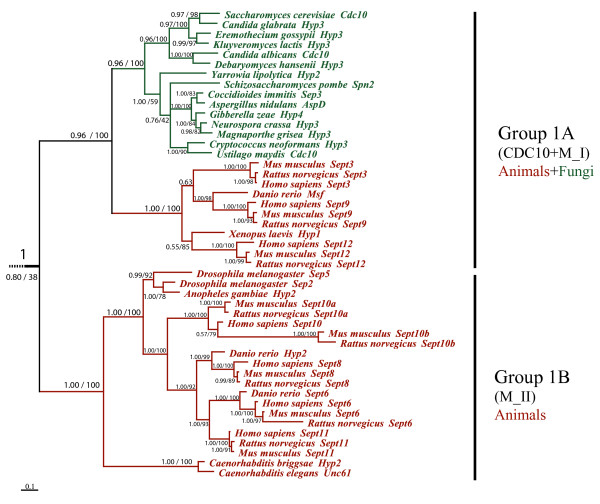
**Group 1 septin phylogenetic tree**. Group 1 from figure 2 enlarged to show species names. Red branches indicate animal lineage and green indicate fungal lineage.

**Figure 4:**
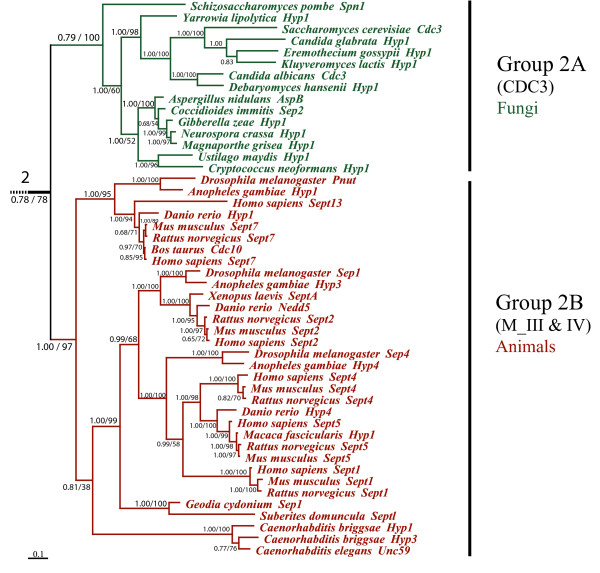
**Group 2 septin phylogenetic tree**. Group 2 from figure 2 enlarged to show species names. Red branches indicate animal lineage and green indicate fungal lineage.

**Figure 5:**
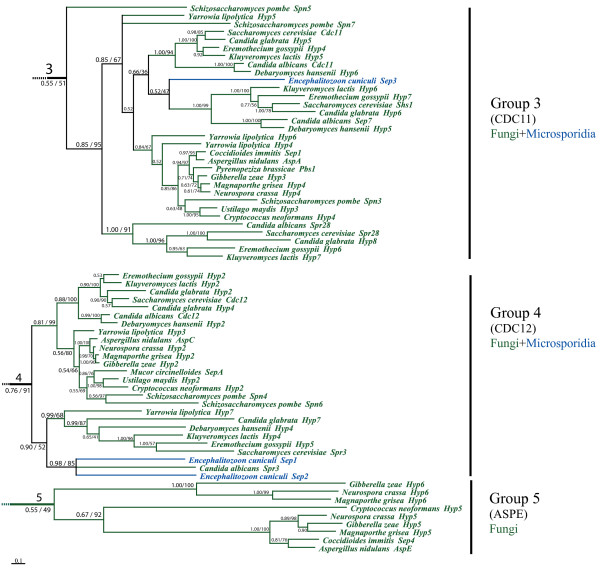
**Groups 3, 4 and 5 septin phylogenetic tree**. Group 1 from figure 2 enlarged to show species names. Green branches indicate fungal lineage and blue indicate microsporidial lineage.

To investigate the evolutionary history of the septin gene family, we used the MrBayes program [29] to construct a phylogenetic tree for all 162 septins, rooting the tree with the *S. cerevisiae *myosin Myo2p. The septins could be grouped into five major clades (Figure 2). Two clades contained fungal and animal septins (Groups 1 and 2) (Figure 3 and Figure 4); two clades contained fungal and microsporidial septins (Groups 3 and 4); one clade contained only fungal septins (Group 5) (Figure 5). Group 1 consisted of two subgroups, 1A and 1B. Subgroup 1A further partitioned into one fungal clade and one animal clade supported by 0.96 credibility. The animal septins in Group 1A were closer to fungal Cdc10-type septins than to other animal septins. Group1A provides the strongest evidence for orthologous relationships between fungal and animal septins, suggesting the ancestral septin that gave rise to members of Group 1A originated before the fungal/animal split. Orthologous relationships between fungal septins in Group 2A and animal septins in Group 2B were supported with 0.78 credibility. Group 3 contained fungal and microsporidial septins. Though the credibility for Group 3 was only 0.55, all sequences except SpoSpn5, fell within a large clade with 0.85 credibility suggesting that the ancestral septin which gave rise to Group 3 arose before the fungal/microsporidial split. Group 4 also contained fungal and microsporidial septins. Though it had a moderate credibility score of 0.76, sequences from Group 4 consistently fell within this clade. The small clade containing microsporidial septins EcuSep1 and EcuSep2 and fungal septin CalSpr3 had 0.98 credibility suggesting that the ancestral septin which gave rise to Group 4 also arose before the fungal/microsporidial split. Group 5, the smallest group, contained septins solely from filamentous fungi. The lack of orthologs from budding or fission yeast suggests that Group 5 septins either arose early in fungal evolution and were lost from yeasts or arose relatively late in fungal evolution.

#### Fungal septins

Ascomycetes with completed genome sequences had five to eight septins while basidiomycetes had four or five (Table 1). All fungi had single Group 1 and Group 2 septins. In contrast, at least some fungi had multiple Group 3, Group 4 and Group 5 septins. In particular, ascomycetous yeasts had three Group 3 septin paralogs. *U. maydis *and *Eremothecium gossypii *are the only two filamentous fungi in our study that lacked a Group 5 septin.

#### Animal septins

In the animals with completed genomes, nematodes had two or three septins, insects had four or five, fish had six and mammals had twelve or thirteen septins (Table 1). All animal septins fell within Groups 1 or 2, with Group 2B often showing the most expansion. The nematode *Caenorhabditis elegans *contained one septin from Group 1B and one from Group 2B. *C. briggsae *also had a single Group1B septin, but contained two Group 2B septins. The insect *Anopheles gambiae *contained a single Group 1B septin and three Group 2B septins, while *Drosophila melanogaster *had an additional Group 1B septin. The zebrafish *Danio rerio *contained a septin from Group 1A along with two septins from Group 1B and three from Group 2B. The mammals *Mus musculus *and *Rattus norvegicus *contained three Group 1A septins and five Group 2B septins. *Homo sapiens *contained three Group 1A septins and six Group 2B septins. *M. musculus *and *R. norvegicus *had five Group 1B septins while *H. sapiens *had four Group1B septins. Martinez and Ware previously divided mammalian septins into groups designated I-IV [6,30,31]; those groups fell within our Groups 1A, 1B and 2B as indicated in Figure 2.

#### Microsporidial septins

*E. cuniculi*, the single microsporidium with a completed genome included in our study, had three septins. *E. cuniculi *contained a single Group 3 sequence and two Group 4 sequences. In contrast to all fungi and animals in our study, *E. cuniculi *contained no sequences from Groups 1 or 2.

#### Validation of tree topology using maximum likelihood

Maximum likelihood non-parametric bootstrapping is not ideal for large datasets; bootstrap values decrease as the taxon number increases [32] and the fast bootstrap methods without branch-swapping typically applied to large datasets may not be reliable at nodes with weak support [33]. None-the-less, because the nodes near the base of our Bayesian tree were weakly supported, we also constructed a phylogenetic tree using maximum likelihood methods. We used the PhyML program with 1024 bootstrap replicates to construct a phylogenetic tree with all 162 septins. The maximum likelihood tree gave the same basic tree topology as the Bayesian tree. For Groups 2, 3 and 5 maximum likelihood support values were similar to Bayesian support values (Figure 2, 78% versus 0.78, 51% versus 0.55 and 49% versus 0.55, respectively). For Group 4 the likelihood support value was higher than the Bayesian value (91% versus 0.76). For Group 1 the likelihood support value was much lower than the Bayesian support value (38% versus 0.8). However, support for Groups 1A and 1B was very similar by both methods (100% versus 0.96 and 100% versus 1.0).

#### Proposed names

Many of the septins we identified are listed as hypothetical proteins in GenBank or have been named after less related septins. We propose to name septins after the most closely related well-characterized septin within the same group (Table 1). The clades upon which proposed names are based are strongly supported and far from the base of the tree (Figure 2). Using this system, fungal and microsporidial septins from Groups 1–4 would be named Cdc3, Cdc10, Cdc11, Cdc12, Shs1, Spr3 or Spr28 after the most closely related *S. cerevisiae *septin and those from Group 5 would be named AspE after the *A. nidulans *septin. The only exception would be fungal septins from *S. pombe*, which would continue to be called Spn1-7 because cell division cycle mutants bearing the Cdc name, but not correlating to the *S. cerevisiae *numbers, have been isolated independently. Mammalian and fish septins from Groups 1 and 2 would be named Sept1-13 after the human septins. Nematode septins would be named Unc59 and Unc61 after the *C. elegans *septins and insect septins would be named Pnut, Sep1, Sep2, Sep4, and Sep5 after *D. melanogaster *septins.

### Domains and Motifs

To identify common motifs, we aligned all 162 septins and analyzed sequence patterns using the Weblogo program [34,35]. In the following sections, septin amino acid positions are referenced to Cdc3p of *S. cerevisiae*.

#### GTPase domains

The G1 motif (GxxxxGK [ST]; SceCdc3 126–133) was the most conserved among the septin motifs (Table 2a). Glycines (G) were found in the first and sixth position in 99%–100% and in the fourth position in 94% of septins. Either K or R occupied the seventh position in 98%. All animal septins, all Group 1A fungal septins and all Group5 septins had a perfect consensus G1 motif. Eight fungal and microsporidial septins from Groups 2, 3 and 4 had derivatives of the consensus G1 motif (see additional file 1). Our analysis also revealed that the two positions immediately following the G1 motif (SceCdc3 134–135) were [TS] [LF] in 96%–97% of septins (Table 2a). A Prosite search using the extended G1 as query also identified other GTPases, so this extended G1 motif is not septin-specific.

**Table 2:** Conserved motifs and single residues in septins.

a. Established Motifs and Extensions				
Amino acid	Frequency (162 total)	Bitscore	Other Residues^+^	

**G1 motif (SceCdc3 126–135)**				

G	162(100%)	4.3	/	
x	/	/	/	
x	/	/	/	
G	152(94%)	3.6	N(4)	
x	/	/	/	
G	160(99%)	4.2	/	
[KR]	158(98%)	3.4/0.4	/	
[ST]	154(95%)	2/1.1	/	
[TS]*	157(97%)	3.4/0.3	A(3)	
[LF]*	156(96%)	1.9/1.2	M(3)	

**G3 motif (SceCdc3 204–209)**				

D	135(83%)	2.7	E(6), N(4), S(4), L(3), T(3)	
T*	141(87%)	3.1	A(7), S(5)	
[PV]*	139(86%)	2.1/0.3	E(6), H(3)	
G	153(94%)	3.8	N(4)	
x*	/	/	/	
G*	150(93%)	3.7	E(6)	

**G4 motif (SceCdc3 280–289)**				

N*	156(96%)	3.8	T(5)	
x*	/	/	/	
x*	/	/	/	
P*	159(98%)	4	L(2)	
x*	/	/	/	
I*	147(91%)	3.3	L(9), V(5)	
x	/	/	/	
K	148(91%)	3.4	R(11)	
x	/	/	/	
D	160(99%)	4	/	

* Modifications of previously defined motifs.				
^+ ^Most common examples of other residues; not all possibilities shown.				

b. New Septin Motifs				

Amino acid	Frequency	Bitscore	Other Residues^+^	

**Sep1 motif (SceCdc3 237–242)**				

E	156(96%)	3.9	D(5)	
x	/	/	/	
x	/	/	/	
x	/	/	/	
x	/	/	/	
R	158(98%)	3.9	T(2)	

**Sep2 motif (SceCdc3 247–259)**				

D	156(96%)	3.9	E(2), G(2)	
x	/	/	/	
R	150(93%)	3.5	H(5)	
[VI]	155(96%)	1.9/1	P(4)	
H	153(94%)	3.6	D(4), Q(4)	
x	/	/	/	
x	/	/	/	
x	/	/	/	
[YF]	156(96%)	3.2/0.3	L(5)	
F	147(91%)	3.3	L(9)	
[IL]	153(94%)	2.9/0.3	V(8)	
x	/	/	/	
P	142(88%)	3.1	A(10), S(3)	

**Sep3 motif (SceCdc3 261–268)**				

G	140(86%)	3	S(6)	
x	/	/	/	
x	/	/	/	
L	151(93%)	3.2	D(5), I(4), V(3)	
x	/	/	/	
x	/	/	/	
x	/	/	/	
D	156(96%)	3.9	E(6)	

**Sep4 motif (SceCdc3 364–365)**				

W	149(92%)	3.7	D(4)	
G	149(92%)	3.6	/	

^+ ^Most common examples of other residues; not all possibilities shown.				

c. Single Conserved Position				

SceCdc3				
Position	Amino Acid	Frequency	Bitscore	Other Residues^+^
117	G	140(86%)	3.3	P(4)
295	[ED]	152(94%)	3.1/0.2	H(5)
300	K	150(93%)	3.6	R(9)
339	P	150(93%)	3.5	D(6)
360	R	150(93%)	3.4	/
396	T	150(93%)	3.4	S(5)

^+ ^Most common examples of other residues; not all possibilities shown.				

The two consensus amino acids in the established GTPase G3 motif (DxxG; SceCdc3 204–207) were found in 83%–94% of septins. Our analysis also showed that the G3 motif consensus for septins could be further modified to DT [PV]GxG (SceCdc3 204–209) with each additional position conserved in 86%–93% of septins (Table 2). Modified G3 motifs were found in all groups except for the animal and fungal Group 1A (see additional file 1).

In the G4 GTPase motif (NKxD, SceCdc3 286–289) N286 was often replaced by A, S, or G. K and D (SceCdc3 287 and 289) were found in 91% and 99% of septins, respectively. Perfect G4 consensus sequences were found in animal Group 1B and fungal Groups 2A and 4 and in fungi in Group 1A. Derived G4 motifs were found in fungal Groups 3 and 5 and in animal members of Group 1A and 2B. We also detected the pattern NxxPxI (SceCdc3 280–285) immediately upstream of the established G4 motif, with each of the three conserved amino acids in 91%–98% of septins. A Prosite search using this extended G4 pattern as query identified other GTPases, so it is not septin-specific.

#### Coiled-coil domains

The coiled-coil is a common structural motif that forms a super helix with heptad repeats and mediates protein-protein interactions [36,37]. It exists in a broad range of proteins involved in numerous cellular processes [38]. Coiled-coil motifs have previously been identified at the C-terminus of the *S. cerevisiae *septins Cdc3 and Cdc12 where they are required for septin association and function [21]. A C-terminal coiled-coil has also been identified in Cdc11, but it is dispensable for function. Cdc10 is shorter than the other *S. cerevisiae *septins and lacks the C-terminal coiled-coil. We analyzed all 162 septin sequences for predicted coiled-coil domains using the COILS [39] and Multicoil programs [40]. Every member of the fungal Group 2A (Cdc3p) and the closely related animal Group 2B contained a predicted coiled-coil domain (Table 1). Similarly, all members of the fungal and microsporidial Group 4 (Cdc12p) contained the predicted coiled-coil. None of the animal or fungal septins in Group 1A (Cdc10p) had a predicted coiled-coil, while all the animal septins in the sister clade Group 1B (M_II) had a predicted coiled-coil. None of the nine septins in the filamentous fungal Group 5 (AspE) were strongly predicted to have the coiled-coil; however, NcrHyp6, MgrHyp6, CneHyp5 and Gzehyp5 had weakly predicted coiled-coil domains (average probability across different window sizes < 0.7, rather than 1). Though most members of the fungal and microsporidial Group 3 (Cdc11p) had a predicted C-terminal coiled-coil, five of the twenty-nine septins in Group 3 had no predicted coiled-coil (EcuSep3, CalSpr28, EgoHyp6, KlaHyp7 and SpoSpn7). Interestingly, theascomycetes that have a Group 3 septin lacking a predicted coiled-coil contain two other Group 3 paralogs with predicted coiled-coils. However, the microsporidium *E. cuniculi *has only a single Group 3 septin.

#### New septin motifs

The Weblogo program assigned bitscores to amino acids in the established G1, G3 and G4 GTPase motifs ranging from a low of 2.7 (SceCdc3 position 204) to a high of 4.3 (SceCdc3 position 126). By considering relative frequency and using positions with bitscores above 2.7, we identified four new septin motifs and designated them Sep1- 4 (Table 2b) and six new conserved single amino acid positions (Table 2c). The Sep1 motif, ExxxxR (SceCdc3 position 237–242) is located between the established G3 and G4 domains (Figure 1) with each of the two consensus amino acids conserved in 96–98% of septins. A Prosite search of the NCBI protein database using the Sep1 motif returned many proteins that were neither septins nor GTPases. The Sep2 motif, DxR [VI]Hxxx [YF]F [IL]xP (SceCdc3 247–259) is located between the G3 and G4 GTPase domains (Figure 1). Each consensus amino acid was present in 88%–96% of septins. A Prosite search with the Sep2 motif identified only septins, but not all septins, making this motif potentially useful for identification of new septin sequences. Four septins with a P rather than a V or I at position 250 are all in the SceSpr28 subclade of Group3. The Sep3 motif, GxxLxxxD (SceCdc3 261–268), is between the G3 and G4 GTPase domains (Figure 1). Each consensus amino acid was present in 86%–96% of septins. A Prosite search with the Sep3 motif returned GTPases including septins. In position 264, the hydrophobic L is often conservatively replaced by I or V. Only members of Group5 have the charged residue D at 264. The Sep4 motif, WG (SceCdc3 364–365), is in the C-terminus within the previously identified "septin unique element" and before the coiled-coil (Figure 1). The amino acids at these two positions were conserved in 92% of septins. A Prosite search with the Sep5 motif showed that it was also found in some other GTPases and hence is not septin-specific.

In addition to the four septin motifs, we detected six positions that contained single consensus amino acids in 86%–94% of septins (Table 2c). One of these positions, upstream of the G1 GTPase motif in the polybasic region (SceCdc3 117; Figure 1), had a G in 99% of animal septins. In fungal septins it was moderately conserved except for four of the Group 5 septins where a P was substituted. The remaining five conserved single amino acid positions were after the G4 motif (SceCdc3 295, 300, 339, 360, and 396). In position 295, 94% of septins had the acidic residues D or E. However, in five septins from Group 5 the basic H residue was substituted.

#### Splice variants

Mammalian septins exhibit complex expression patterns and can produce a large number of splicing variants [28]. The human septin, SEPT9, spans a 240 kb region, contains 17 exons, and is predicted to have 18 different transcripts encoding 15 polypeptides [41]. All of the conserved positions identified in our study were predicted to be retained in all variants encoded by SEPT9. Indeed, all splicing of human septin transcripts so far reported occurs in the regions encoding N- or C- termini and not in the regions encoding the conserved core of the protein.

## Discussion

### Evolution

The origin of the septins in eukaryotes depends upon the interpretation of the septin-like sequence we found in *Giardia lamblia*. If this is considered a primitive septin, then a septin-like ancestor existed before the diplomonads arose. This septin-like ancestor was retained in the diplomonads, animals, fungi and microsporidia, but lost in plants. If the *G. lamblia *septin-like sequence is part of a separate GTPase family that shares some motifs with septins, then septins may have entered the common ancestor of animals, fungi and microsporidia via a horizontal gene transfer from bacteria, as proposed by Leipe [10].

**Figure 6:**
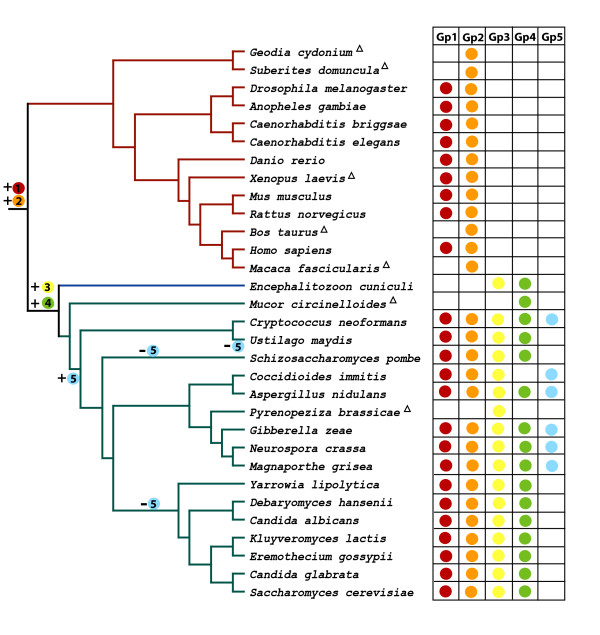
**Postulated septin evolution**. Summary phylogeny of 31 species used in this study. This tree summarizes the evolution of the septins in the 31 organisms whose septins were identified and used in this study [50–53]. Red branches indicate animal lineages and green branches indicate fungal lineages. The table on the right of the tree indicates different groups of septin genes. Group 1 is red, group 2 is orange, group 3 is yellow, group 4 is green, and group 5 is blue. A triangle means the complete genome sequence was not available when the initial search was executed, so some septins might not have been identified due to incomplete sequence information.

Which ever origin is correct, our phylogenetic analysis suggests that septins might have evolved as follows (Figure 6): The ancestral septin sequence duplicated before the divergence of animals and fungi to become the ancestral Group 1 and Group 2 septins. The ancestral Group 1 septin duplicated and one paralog lost the C-terminal coiled-coil extension. Animals and fungi retained this shortened Group 1 paralog which gave rise to Group 1A septins. The longer paralog containing the C-terminal extension was lost from fungi, but retained in animals giving rise to Group 1B septins. Within fungal species there is a single Group 1 paralog, however in many animals, especially mammals, extensive duplication gave rise to multiple Group 1 paralogs. The ancestral Group 2 septin was retained in both animals and fungi giving rise to Group 2A and Group 2B septins. Fungi have single paralogs of Group 2 septins, while most animals, especially mammals, have multiple paralogs. In the lineage leading to fungi and microsporidia, the ancestral Group 1 and Group 2 septins duplicated giving rise to Group 3 and Group 4 septins. Unlike the single fungal paralogs of Group 1 and Group 2, Group 3 and Group 4 septins duplicated and diverged, giving rise to multiple paralogs, especially in the ascomycetes. In the lineage leading to microsporidia, Group 1 and Group 2 septins were lost. This is consistent with recent views that microsporidia evolved from fungi [42]. Group 5 septins, found only in filamentous fungi, either arose early in fungal evolution and were lost in yeasts or arose relatively recently.

### Motifs

#### Polybasic domain and Septin element

To be considered septin motifs, we required that sequences be at least as conserved as the GTPase motifs. While this stringent cut-off undoubtedly eliminated moderately-conserved or clade-specific sequences, it guaranteed the significance of identified positions. Only one amino acid (SceCdc3 117G) within the ten amino acid polybasic region previously shown to bind phosphoinositides (Figure 1; SecCdc3 110–120) [20] was conserved enough across all septins to be considered a septin motif in our analysis. Similarly, only 6 amino acids (sep1 motif and 2 conserved single amino acid positions) within the previously defined 53 amino acid "septin unique element" (SceCdc3 360–413) [23] meet our cut-off for septin motifs.

#### GTPase

Septins have been shown to bind and hydrolyze GTP [23]. Many lines of evidence suggest that guanine-nucleotide binding by septins is needed for their polymerization; however, low rates of nucleotide exchange and hydrolysis *in vitro *have led to questions about the significance of the GTPase activity. Consistent with the importance of guanine nucleotide binding for septin function, our analysis showed that the G1 GTPase motif, which forms the loop that interacts with the phosphate group of the nucleotide, and the G4 motif, which is important for GTP-binding specificity, were highly conserved, with 154 of 162 (95%) septins matching the respective consensus sequences (see additional file 1). In contrast the G3 motif, which binds to the Mg2+ ion, matched the consensus for 135 of 162 (83%) septins.

#### Coiled-coil

In *S. cerevisiae *all septins except for Cdc10p (Group 1A) are predicted to have a C-terminal region containing a coiled-coil, a motif implicated in protein-protein interactions. Like Cdc10p, all Group 1A septins are missing the C-terminal region that contains the coiled-coil. Group 1B septins are all predicted to contain C-terminal coiled-coils. In elegant work, Versele and Thorner [21] showed that *S. cerevisiae *Cdc3p and Cdc12p associate through their C-termini and that Cdc11p and Cdc12p associate independently of their C-termini. In our analysis all Group 2 (Cdc3p) and Group 4 (Cdc12p) septins were predicted to contain C-terminal coiled-coils, while 5 of 29 Group 3 (Cdc11p) septins were not predicted to contain C-terminal coiled-coils. This pattern of conservation suggests that C-terminal coiled-coil interactions might be important for the association of all Group 2 (Cdc3p) septins with Group 4 (Cdc12p) septins while interactions outside the C-terminus might be important for the association of all Group 2 with Group 3 septins. Animals lack Group 4 septins, but Group 1B septins likely play the same role in polymerization by interacting with Group 2 septins. Indeed, mammalian Sept6 (Group 1B) and Sept7 (Group 2B) have been shown to interact via their C-termini leading Versele and Thorner to suggest that the Sept6–Sept7 complex is the animal counterpart of the Cdc3p-Cdc12p complex [23]. Group 5 septins, found in filamentous fungi, lack or have weakly predicted coiled-coils, suggesting that C terminal regions are not important for their interactions.

## Conclusion

We analyzed 162 septins from microsporidia, fungi and animals. Septins were grouped into five classes, modified nomenclature based on these five classes was suggested and there was strong evidence for orthology between septins from different kingdoms. In addition to derivatives of already known G1, G3 and G4 GTPase motifs, four new motifs and six conserved single amino acid positions were identified. Though first discovered and best-studied in the yeast *S. cerevisiae *it has become increasingly clear that the septins are important in animals. Earlier work based on septins from only five species suggested that there were no clear orthologs between the septins in fungal systems and those in mammals [25] confounding extrapolation from simple to more complex systems. With the availability of many more sequences, our work clarifies the relationships among septins and points to which comparisons are likely to be most informative.

## Methods

### Database searches

We used the 520-residue *Saccharomyces cerevisiae *septin protein Cdc3p (GenBank: gi|2507385) as the initial query sequence for PSI-BLAST searches against the non-redundant database (All non-redundant GenBank CDS translations+RefSeq Proteins+PDB+SwissProt+PIR+PRF) at NCBI [43]. PSI-BLAST performs iterative profile searches by generating position specific scoring matrices to achieve high sensitivity. Three iterations were run with default parameters (Expect Value 10, Word Size 3, Blosum62, Gap Opening Penalty 11, Gap Extension Penalty 1, and With Inclusion Threshold 0.005) until no new septin or septin-like sequences were found. We examined each sequence retrieved from the PSI-BLAST output and removed duplicated and obviously incomplete sequences. We classified the remaining sequences as septins or septin-like proteins by examining the three GTP motifs of septins [25]: G1 (GxxxxGK [S/T]), G3 (DxxG) and G4 (xKxD) and their phylogenetic relationships with other septins.

### Protein alignments

We used CLUSTALX1.8 for protein multiple sequence alignment [44]. Default parameters were used, as no significant differences were observed when we tested different parameter combinations. Protein weight matrix Gonnet 250, with Gap Opening Penalty 10 and Gap Extension Penalty 0.1 was used for pairwise alignments. Protein weight matrix Gonnet, with Gap Opening 10 and Gap Extension 0.2 was used for multiple alignments. We manually modified the multiple alignment output from ClustalX with the Bioedit program [45]. We used Weblogo Version 2.8.1 to show the consensus structure of the sequences [34,35]. Bit scores from the output were also used to help identify conserved regions.

### Reconstruction of phylogenetic trees

We used MrBayes v3.1 for phylogenetic analysis [29]. The amino acid model was estimated using the setting "aamodelpr = mixed" allowing the program to test and use the best fitting model for the dataset from 9 fixed rate protein models. We used 1,500,000 running generations, sample frequency of 200 and burn in period set to 40,000 to keep only the stationary phase samples. The chain number was set to 4 with 1 cold chain and 3 heated chains with heating coefficient λ = 0.2. Two independent analyses were run simultaneously and converged. The consensus type was set to halfcompact. The myosin sequence from *Saccharomyces cerevisiae *Myo2p (gi|6324902) was used as outgroup. We also used PhyML [46] for maximum likelihood with bootstrap analysis of 1,024 replicates. The JTT amino acid substitution model was used. The proportion of invariant sites was estimated by maximizing the phylogeny likelihood. The number of relative substitution rate categories was set to 4 with gamma distribution parameter equal to 1. Tree topology, branch lengths and rate parameters were optimized.

### Domain and secondary structure predictions

We checked each sequence for domains with the Simple Modular Architecture Research Tool [26,47]. An NCBI Conserved Domain Search was also used [48]. Sequences were searched for coiled-coil domains with the COILS program [39]; default parameters were used. Results from Multicoil were also considered [40]. Sequences with average probability above 0.7 were considered to have coiled-coil domains. Secondary structure was predicted using PSIPRED [49].

## Authors' contributions

FP carried out the analysis and drafted the manuscript. RLM participated in the design of the study, helped in the analysis and helped to draft the manuscript. MM participated in the design of the study, helped in the data interpretation and helped to draft the manuscript and revise it critically. All authors read and approved the final manuscript.

## Supplementary Material

Additional file 1 Septin Derived GTPase Motifs. Click here for file
