# Supplementary material for: Analysis of septins across kingdoms reveals orthology and new motifs
Source: BMC Evol Biol. 2007 Jul 1;7:103. doi: 10.1186/1471-2148-7-103 (PMC1931588; doi:10.1186/1471-2148-7-103)
Supplement: Additional file 1 — Septin Derived GTPase Motifs. [file 1471-2148-7-103-S1.doc]

**Supplementary Table 1. Septin Derived GTPase Motifs***

| a. G1 GTPase Domain: GxxxxG[KR][ST] | | |
| --- | --- | --- |
| **Groups** | **Sequence** | **Derived Motifs** |
| 2A | CalCdc3 | GesglGKA |
| 3 | CalSpr28 | GvndlGKK |
| 2A | DhaHyp1 | GesglGKK |
| 4 | EcuSep2 | GrrglGTS |
| 3 | NcrHyp4 | GasgtGES |
| 3 | PbrPbs1 | GsslsPLV |
| 3 | SpoSpn7 | GssytSYQ |
| 3 | YliHyp6 | GpggsGRA |

| b. G3 GTPase Domain: DxxG | | |
| --- | --- | --- |
| **Groups** | **Sequences** | **Derived Motifs** |
| 3 | CalCdc11 | DtpN |
| 2A | CalCdc3 | TapG |
| 3 | CalSpr28 | VtnN |
| 4 | CalSpr3 | EtvN |
| 4 | CglHyp2 | DtpA |
| 3 | CglHyp6 | MtlG |
| 3 | CglHyp8 | ImeG |
| 2A | DhaHyp1 | StpG |
| 3 | DhaHyp6 | DtpN |
| 2B | DreHyp1 | (missing) |
| 1B | DreSept6 | NtvG |
| 4 | EcuSep2 | TyhE |
| 3 | EgoHyp6 | LapG |
| 5 | GzeHyp6 | TrkR |
| 1B | HsaSept10 | NtvG |
| 1B | HsaSept6 | StvG |
| 3 | KlaHyp7 | LipG |
| 1B | MmuSept10a | NtvG |
| 1B | MmuSept10b | KtvG |
| 1B | MmuSept6 | StvG |
| 1B | RnoSept10a | NtvG |
| 1B | RnoSept10b | KtvG |
| 1B | RnoSept6 | StvG |
| 3 | SceShs1 | MthG |
| 3 | SceSpr28 | LfpG |
| 3 | SpoSpn7 | EvnG |
| 3 | YliHyp5 | EgpG |

| c. G4 GTPase Domain: xKxD**^†^** | | |
| --- | --- | --- |
| **Groups** | **sequence** | **Derived Motifs** |
| 5 | CneHyp5 | sNvE |
| 5 | GzeHyp6 | aRaD |
| 1A | HsaSept12 | aRaD |
| 1A | Mmusept12 | aRaD |
| 5 | NcrHyp6 | sQaD |
| 1A | RnoSept12 | aRaD |
| 2B | SdoSept1 | iKcP |
| 3 | SpoSpn7 | gNsN |

*Upper case represents previously identified conserved positions.

† Loss of asparagine in the G4 motif NKxD is a feature for septin family, so the motif is represented by xKxD.
